# Development of Multienzyme Isothermal Rapid Amplification (MIRA) Combined with Lateral-Flow Dipstick (LFD) Assay to Detect Species-Specific *tlh* and Pathogenic *trh* and *tdh* Genes of *Vibrio parahaemolyticus*

**DOI:** 10.3390/pathogens13010057

**Published:** 2024-01-06

**Authors:** Seong Bin Park, Yan Zhang

**Affiliations:** Experimental Seafood Processing Laboratory, Coastal Research & Extension Center, Mississippi State University, Pascagoula, MS 39567, USA

**Keywords:** *Vibrio parahaemolyticus*, *tlh*, *trh*, *tdh*, oyster, MIRA, LFD

## Abstract

*Vibrio parahaemolyticus* causes severe gastroenteritis in humans after consuming contaminated raw or undercooked seafood. A species-specific marker, the thermolabile hemolysin (*tlh*) gene, and two pathogenic markers, thermostable-related hemolysin (*trh*) and thermostable-direct hemolysin (*tdh*) genes, have been used to identify *V. parahaemolyticus* and determine its pathogenicity using both PCR and qPCR assays. To enable testing in field conditions with limited resources, this study aimed to develop a simple and rapid method to detect the species-specific (*tlh*) and pathogenic (*trh* and *tdh*) genes of *V. parahaemolyticus* using multienzyme isothermal rapid amplification (MIRA) combined with a lateral-flow dipstick (LFD). The amplification of the *tlh*, *trh*, and *tdh* genes could be completed within 20 min at temperatures ranging from 30 to 45 °C (*p* < 0.05). The test yielded positive results for *V. parahaemolyticus* but produced negative results for nine *Vibrio* species and eighteen foodborne pathogenic bacterial species. MIRA-LFD could detect 10 fg of DNA and 2 colony-forming units (CFU) of *V. parahaemolyticus* per reaction, demonstrating a sensitivity level comparable to that of qPCR, which can detect 10 fg of DNA and 2 CFU per reaction. Both MIRA-LFD and qPCR detected seven *tlh*-positive results from thirty-six oyster samples, whereas one positive result was obtained using the PCR assay. No positive results for the *trh* and *tdh* genes were obtained from any oyster samples using MIRA-LFD, PCR, and qPCR. This study suggests that MIRA-LFD is a simple and rapid method to detect species-specific and pathogenic genes of *V. parahaemolyticus* with high sensitivity.

## 1. Introduction

*Vibrio parahaemolyticus* is a Gram-negative halophilic bacterium that inhabits warm estuarine and marine environments worldwide [1]. It has been isolated from various fish and shellfish species, earning it a reputation as one of the critical foodborne pathogenic bacteria responsible for illness after consuming contaminated raw or undercooked seafood [2]. The U.S. Centers for Disease Control and Prevention (CDC) estimates that approximately 84,000 people have suffered from foodborne illnesses due to *Vibrio* infections in the U.S., even though most *Vibrio* infections are not officially recorded [3,4].

Two distinctive pathogenic markers, namely, the thermostable-related hemolysin encoded by the *trh* gene and thermostable-direct hemolysin encoded by the *tdh* gene, have been recognized as crucial indicators of the pathogenicity of *V. parahaemolyticus*-induced gastroenteritis [5,6,7,8]. Despite sharing a 67% similarity in their amino acid sequences and having similar predicted functions, both genes serve as essential pathogenic markers [9]. A study has revealed that clinical isolates demonstrate over 80 percent *tdh* positivity and less than 20 percent *trh* positivity [10]. Similarly, more than 90 percent of patients suffering from *V. parahaemolyticus* infections have shown *tdh* positivity [11]. However, *V. parahaemolyticus* isolates from environmental sources are generally considered avirulent to humans, as only a low number of isolates showed *trh* or *tdh* positivity [10,12]. For instance, an investigation conducted in the coastal areas of Georgia and South Carolina in the U.S. demonstrated that only 0.3% and 4.3% of *V. parahaemolyticus* strains possessed the *trh* and *tdh* genes, respectively [13]. As almost 90% of *V. parahaemolyticus* infections stem from eating undercooked or raw oysters in the U.S. [4], identifying pathogenicity (*trh* and/or *tdh* positivity) in *V. parahaemolyticus* is of utmost importance, not only to ensure public health but also to protect the seafood industry.

Although various genes have been selected for identifying *V. parahaemolyticus*, including *tlh*, pR72H, *toxRS*, *toxR*, and ORF8 [14,15,16,17,18], the *tlh* gene is currently used as the species-specific marker to identify *V. parahaemolyticus* according to the *Bacteriological Analytical Manual* (BAM) published by the U.S. Food and Drug Administration (FDA) [19]. This gene encodes the thermolabile hemolysin, which has not been reported to show virulence in humans, while most virulent isolates demonstrated *trh* and/or *tdh* positivity [2,14]. Various PCR and qPCR methods have been introduced to confirm *tlh*, *trh*, and *tdh* positivity, showing high sensitivity and specificity [20]. For instance, a multiplex PCR assay could detect *tlh*, *trh*, and *tdh* positivity from 100 CFU/10 g of oyster homogenates [14], while a multiplex qPCR assay could detect those genes from less than 10 CFU/reaction of *V. parahaemolyticus* [21]. Both assays showed high specificity, with no positive reactions from other *Vibrio* species or foodborne bacteria.

The recombinase polymerase amplification (RPA) assay has been proposed for the rapid identification of various pathogens in restricted field conditions, as PCR assays require laboratory conditions, including well-trained operators and PCR-related instruments [22,23,24]. RPA is an isothermal nucleic acid amplification method that completes target gene amplification via an enzymatic primer–protein binding process within 15 min at 37–42 °C [25]. The subsequent RPA amplicon can be visualized by using the lateral-flow dipstick (LFD) assay, enabling the result to be shown by the visualization of both the control line and positive test line on a strip within 5 min [26]. So far, four RPA detection methods have been developed to detect *V. parahaemolyticus* in seafood using the *gyrB*, NC_004605, *ToxR*, and *tlh* genes, and studies indicate that RPA-LFD is a promising method to use in field conditions [27,28,29,30]. However, there has been no RPA research on the development of two distinctive pathogenic markers (*trh* and *tdh*) of *V. parahaemolyticus,* while a study demonstrated the difficulty of developing RPA for both genes [30].

Recently, a new RPA method, known as multienzyme isothermal rapid amplification (MIRA), has been introduced to achieve the rapid, sensitive, and specific amplification of the target gene through the synergetic action of various functional proteins, including helicase, recombinase, single-stranded binding protein, and DNA polymerase [31]. MIRA uses a different source of recombinase (*Streptomyces azure* recA, SC-recA), which may enhance the stability of the reaction and resistance to interference [31]. In the present study, the MIRA-LFD assay was developed to rapidly detect not only the species-specific *tlh* gene but also the two pathogenic *trh* and *tdh* genes of *V. parahaemolyticus* for field conditions with limited resources. The sensitivity and specificity of the MIRA-LFD assay were determined and compared with those of the PCR and qPCR assays.

## 2. Materials and Methods

### 2.1. Bacterial Strains and DNA Extraction

*Vibrio parahaemolyticus* F11-3A served as the reference strain to determine the amplification of the *tlh*, *trh*, and *tdh* genes [21]. To assess the specificity of RPA-LFD, closely related *Vibrio* strains and other foodborne bacteria were examined (Table 1). All bacteria were cultured on tryptic soy agar (TSA, Remel, San Diego, CA, USA) or in tryptic soy broth (TSB, Remel) at 37 °C. Genomic DNA was extracted from the bacteria using the Quick-DNA Fungal/Bacterial Miniprep Kit (Zymo Research, Irvine, CA, USA) following the manufacturer’s instructions. The concentration of genomic DNA was measured using a NanoDrop spectrophotometer (Thermo Fisher Scientific, Waltham, MA, USA), and DNA samples with spectrophotometric ratios of 1.8 to 2.0 (A260/A280) were stored at −20 °C until use.

### 2.2. Primers and Probes

The *tlh* (Gene ID: 1190914), *trh* (GenBank: KP836460.1), and *tdh* (Gene ID: 1192010) genes were used to design primers and probes using Primer-BLAST (http://www.ncbi.nlm.nih.gov/tools/primer-blast/, accessed on 17 March 2023), Oligo Calc: Oligonucleotide Properties Calculator (http://biotools.nubic.northwestern.edu/OligoCalc.html, accessed on 17 March 2023), and Primer3 Plus (http://www.bioinformatics.nl/cgi-bin/primer3plus/primer3plus.cgi, accessed on 17 March 2023). The 5′ ends of reverse primers were labeled with biotin for attachment to the lateral-flow dipstick (LFD, HybriDetect 1, Milenia Biotec, Giessen, Germany). In addition, the LFD probe was labeled with a polymerase extension blocking group (C3 spacer) at the 5′ end, an internal abasic nucleotide analog (dSpacer tetrahydrofuran residue) replacing a nucleotide, and a carboxyfluorescein (FAM) group at the 3′ end. All primers and probes used for basic RPA and MIRA-LFD in this study are listed in Table 2.

### 2.3. Basic Recombinase Polymerase Amplification (Basic RPA)

To determine the optimal primer set, the basic RPA assay (TwistDx, Cambridge, UK) was conducted using the primers listed in Table 2. In brief, the reaction mixture consisted of 2.4 µL of each primer (10 µM), 29.5 µL of rehydration buffer, 11.2 µL of water, and 2 µL of genomic DNA. This mixture was transferred to a tube containing a lyophilized reaction pellet and mixed by vortexing until the entire pellet was fully resuspended. Following the addition of 2.5 µL of MgAc (280 mM), the reaction was incubated at 40 °C for 20 min using a water bath. The resulting amplicon was then electrophoresed through a 1% TBE (TBE, Alfa Aesar, Ward Hill, MA, USA) agarose gel containing the SYBR Safe DNA gel stain (Invitrogen, Waltham, MA, USA) and visualized using the Gel Doc XR+ system (Bio-Rad, Hercules, CA, USA).

### 2.4. Multienzyme Isothermal Rapid Amplification (MIRA) and Lateral-Flow Dipstick (LFD)

**Figure 1:**
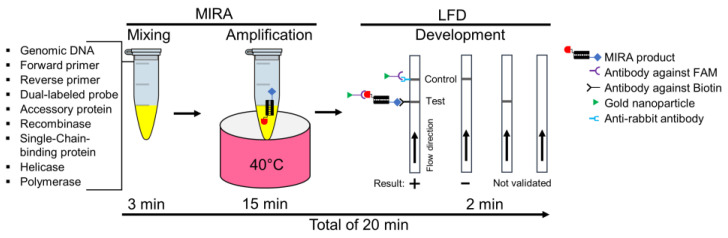
The schematic and workflow of MIRA-LFD for detecting *tlh*, *tdh*, and *trh* genes of *Vibrio parahaemolyticus*. This assay consists of three steps—mixing, amplification, and development—all completed within 20 min. During the MIRA procedure, the amplicon is labeled with FAM and biotin through the reaction of primers, probe, accessory protein, recombinase, single-chain binding protein, helicase, and recombinase. The LFD has both a test line that reacts with the biotin of the amplicon and a control line containing an anti-rabbit antibody that reacts with an antibody conjugated with a gold nanoparticle.

As depicted in Figure 1, the MIRA-LFD assay was carried out by combining the MIRA nfo kit (Amp-Future, Changzhou, China) with the lateral-flow dipstick (LFD). Briefly, a MIRA mixture composed of 11.5 µL of nuclease-free water, 2 µL of DNA template, 29.4 µL of A buffer, 2 µL of forward and reverse primers (10 µM), and 0.6 µL of the probe (10 µM) was added to a test tube containing a lyophilized pellet. After adding 2.5 µL of MgAc (280 mM), the tube was incubated at 40 °C for 15 min to conduct the MIRA reaction. To visualize the amplified product, which was labeled with carboxyfluorescein (FAM) and an antigenic tag (biotin), 5 µL of the MIRA product was diluted in 195 µL of HybriDetect assay buffer. The sample pad of the LFD was immersed in the diluted solution and incubated for 2 min at room temperature. The clear visualization of both test and control lines on the LFD was considered a positive result, while the appearance of only the control band on the strip was regarded as negative.

### 2.5. Optimization of MIRA-LFD

Genomic DNA from *V. parahaemolyticus* F11-3A (1 ng per reaction) was used to determine the optimization of MIRA-LFD. Various incubation temperatures for MIRA were tested, including 25 °C, 30 °C, 35 °C, 40 °C, 45 °C, and 50 °C, with an incubation duration of 15 min. The optimal incubation durations for both MIRA and LFD were determined at a constant temperature of 40 °C. The MIRA step durations tested were 2.5, 5, 10, 15, and 20 min, while the LFD step durations tested were 1.5, 3, 5, and 10 min.

### 2.6. Specificity and Sensitivity of MIRA-LFD

The specificity of MIRA-LFD was validated by applying genomic DNA extracted from various *Vibrio* and foodborne bacterial species listed in Table 1. To determine the sensitivity of MIRA-LFD, both 10-fold serially diluted genomic DNA (ranging from 1 ng to 1 fg per reaction) and direct cultures of *V. parahaemolyticus* F11-3A (ranging from 2 × 10^4^ to 2 CFU per reaction) were employed. Additionally, fresh oysters were seeded with various concentrations of *V. parahaemolyticus* to assess the sensitivity of MIRA-LFD. In brief, fresh oysters were shucked, pooled, blended, and then diluted with phosphate-buffered saline (PBS, pH 7.4) at a ratio of 1:4 (*W*/*V*). After confirming the absence of *V. parahaemolyticus* contamination using a conventional PCR assay (described below), the oyster samples were mixed with various concentrations of F11-3A (ranging from 1.8 × 10^4^ to 1.8 CFU per reaction) for the MIRA-LFD assay.

### 2.7. Comparison of Vibrio Parahaemolyticus Detection in Fresh Oysters by RPA-LFD, PCR, and qPCR

Fresh oysters were procured from local markets in the Northern Gulf States, including Alabama, Florida, Louisiana, and Mississippi, between July and October. Ten oysters were aseptically shucked and blended, and 10 mL of the oyster blend was inoculated in 90 mL of alkaline peptone water (APW) and incubated overnight for enrichment. A total of 36 enrichment samples were employed for the comparative analysis of the detection capabilities of MIRA-LFD, PCR, and qPCR. Two microliters of each sample were directly utilized in the PCR, qPCR, and MIRA-LFD assays.

### 2.8. Polymerase Chain Reaction (PCR) and Quantitative Polymerase Chain Reaction (qPCR)

PCR was conducted to determine assay sensitivity using various genomic DNA concentrations and CFU per reaction, following the *BAM* guidelines of the U.S. FDA with minor modifications [14,19]. In brief, the PCR mixture included 2.5 U of DreamTaq Green DNA polymerase (Thermo Scientific, Vilnius, Lithuania), 10 mM dNTP (2.5 mM each), 1 × PCR buffer, 2 µL of the primer set (10 µM), and 2 µL of the template. The final volume of the mixture was adjusted to 25 µL with water. The amplification conditions for the *tlh* and *tdh* genes were 1 cycle at 94 °C for 10 min, followed by 30 cycles of 94 °C for 1 min, 58 °C for 1 min, and 72 °C for 2 min, with a final extension at 72 °C for 10 min. For the *trh* gene, the PCR conditions were 1 cycle at 94 °C for 3 min, followed by 30 cycles of 94 °C for 1 min, 60 °C for 1 min, and 72 °C for 2 min, with a final extension at 72 °C for 3 min. The amplified PCR product was visualized on 1% tris-borate–EDTA agarose gel containing SYBR Safe DNA gel stain.

The qPCR assay was carried out based on a previous study with minor modifications to validate the detection limits of both bacterial DNA and CFU [21]. For *tlh* amplification, each 25 µL reaction mixture contained 1 × PrimeTime Gene Expression Master Mix (Integrated DNA Technologies, Coralville, IA, USA), 75 nM of each primer (*tlh* 884F, *tlh* 1091R, IAC 46F, and IAC 186R), and 150 nM of *tlh* and IAC probes. For *tdh* and *trh* amplification, each 25 µL reaction mixture contained the 1 × PrimeTime Gene Expression Master Mix, 200 nM of each primer (*tdh* 89F, *tdh* 321R, *trh* 20F, *trh* 292R, IAC 46F, and IAC 186R), 150 nM of IAC probes, and 75 nM of *tdh* and *trh* probes. The reactions contained various concentrations of templates and IAC DNA. The cycling consisted of an initial denaturation at 95 °C for 60 s, followed by 45 cycles of denaturation at 95 °C for 5 s and annealing at 59 °C for 45 s. The signal amplification (ΔRn) was plotted against qPCR cycles, where amplification is detected as exceeding an arbitrary threshold. The primers and probes used for PCR or qPCR are listed in Table 3.

### 2.9. Statistical Analysis

The intensity of the test band was quantified using Image Lab Software Version 6.0.1 (Bio-Rad, Hercules, CA, USA) and was displayed as the relative band intensity in comparison with the value of the negative control. Statistical analyses were carried out using Prism Version 9 (GraphPad, Boston, MA, USA). Significant differences were determined using an ordinary one-way ANOVA analysis (Dunnett’s multiple-comparisons test, *p* < 0.05), and data are presented as mean ± standard deviation (SD, *n* = 3).

## 3. Results and Discussion

### 3.1. Primer Selection for tlh, trh, and tdh Genes for the Application of MIRA-LFD

**Figure 2:**
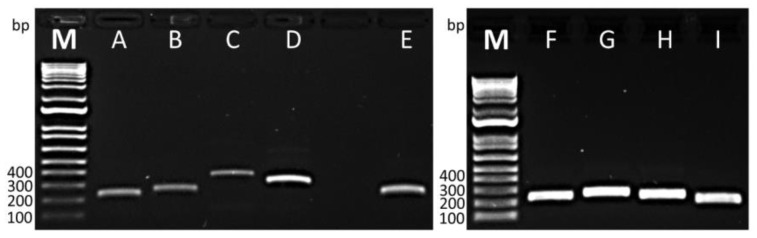
Validation of primers for the amplification of *tlh* (A–D), *tdh* (E), and *trh* (F–I) genes using basic RPA. (A) VP_TLH_F1 and R1 (226 bp), (B) VP_TLH_F1 and R1-2 (256 bp), (C) VP_TLH_F2 and R2 (369 bp), (D) VP_TLH_F2 and R2-2 (312 bp), (E) VP_TDH_F1 and R1 (238 bp), (F) VP_TRH-F1 and R1 (243 bp), (G) VP_TRH-F1 and R1-2 (266 bp), (H) VP_TRH-F2 and R2 (243 bp), and (I) VP_TRH-F2 and R2-2 (208 bp). “M” is an abbreviation for the molecular-weight size marker.

To identify the optimal primer set for the *tlh*, *trh*, and *tdh* genes, we evaluated four combinations (two forward and two reverse), one combination (one forward and one reverse), and four combinations (two forward and two reverse) of primer candidates using basic RPA (Table 1). The basic RPA procedure was conducted in a 40 °C water bath for 20 min, followed by electrophoresis of the RPA products through a 1% TBE agarose gel. Figure 2 illustrates the successful amplification of target amplicons for all primers designed for the *tlh* (A to D), *tdh* (E), and *trh* (F to I) genes. Despite prior research reporting the failure of eight primer pairs targeting the *tdh* and *trh* genes to produce specific amplicons [30], our designed primers demonstrated robust amplification. Our primer design adhered to specific criteria: (1) a primer size ranging from 30 to 36 bp, (2) primer GC% ranging from 20 to 70%, (3) a primer temperature ranging from 50 to 100 °C, and (4) a maximum allowable length of a nucleotide repeat set to 5, following the manufacturer’s recommendations (https://www.twistdx.co.uk/docs/default-source/RPA-assay-design/twistamp-assay-design-manual-v2-5.pdf, accessed on 17 March 2023). This approach is intended to yield clear, specific amplification bands without obvious primer dimers. The primer pair VP_TLH_F1 and VP_TLH_R1 (Figure 2A) was chosen for the *tlh* gene, as it amplified the shortest amplicon size, reducing the risk of primer noise occurrence, as recommended by the manufacturer. For *tdh* gene amplification, the pair VP_TDH_F1 and VP_TDH_R1 (Figure 2E) was employed, and VP_TRH_F1 and VP_TRH_R1 (Figure 2F) were selected for detecting the *trh* gene, as the resulting amplicon exhibited a GC content between 40% and 60%. Importantly, all primer pairs showed no cross-amplification when tested with the bacterial gDNA listed in Table 2.

### 3.2. Optimization of MIRA-LFD to Amplify tlh, trh, and tdh Genes

**Figure 3:**
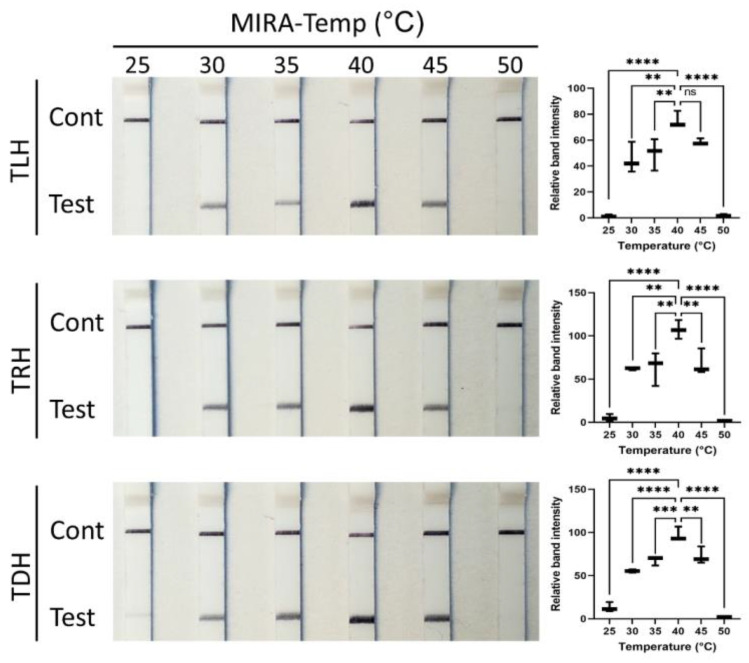
Optimization of temperature (from 25 °C to 50 °C) for *tlh*, *trh*, and *tdh* genes using MIRA. MIRA reactions were conducted for 15 min, and LFD assays were performed for 1.5 min. LFD strips exhibited an upper control line and a lower test line when the MIRA reaction was successfully accomplished. Data represent the means of three independent replicates (one-way ANOVA, ** *p* < 0.01, *** *p* < 0.001, and **** *p* < 0.0001).

MIRA-LFD consists of two procedures: gene amplification using MIRA and the visualization of the test result with an LFD. The incubation temperature and time for MIRA, as well as the incubation time for the LFD, were examined to determine the optimal conditions for detecting the *tlh*, *tdh*, and *trh* genes of *V. parahaemolyticus*. As shown in Figure 3, the MIRA steps for all *tlh*, *tdh*, and *trh* genes could be accomplished in the temperature range of 30–45 °C. No test lines were observed for any of the genes at 25 and 50 °C. MIRA performed at 40 °C exhibited the highest band intensity among the temperatures examined for all three genes (*p* < 0.05). Similarly, RPA for *Salmonella* spp. showed a positive test line in the temperature range of 30–45 °C and displayed the highest test band intensity at 40 °C [32]. However, MIRA for *Acinetobacter baumannii* could amplify at 25 °C and 50 °C [33], and MIRA for *Spiroplasma eriocheiris* did not show amplification even at 30 °C and 40 °C [34]. Taken together, these studies indicate that the optimal temperature range of the MIRA assay is different depending on the templates, primers, and probes; therefore, the optimization of the temperature is a crucial step for the successful detection of target genes through the MIRA assay.

**Figure 4:**
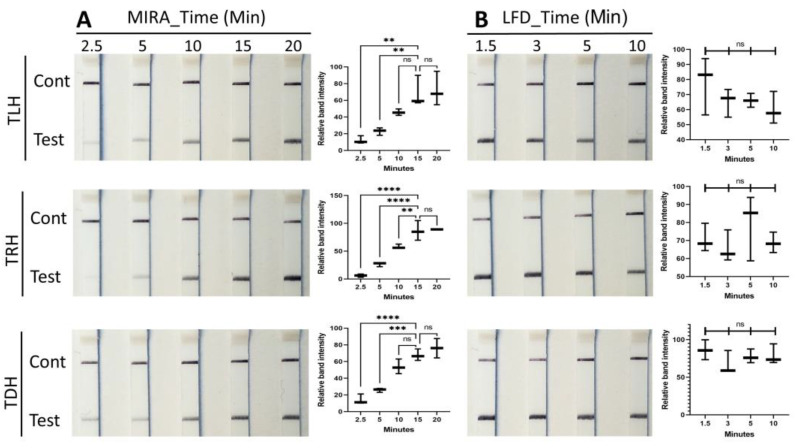
Optimization of MIRA and LFD incubation time for *tlh*, *trh*, and *tdh* genes. MIRA reactions (**A**) were conducted at 40 °C, and LFD assays (**B**) were performed at room temperature. LFD strips exhibited an upper control line and a lower test line when the MIRA reaction was successfully accomplished. Data represent the means of three independent replicates (one-way ANOVA, ** *p* < 0.01, *** *p* < 0.001, and **** *p* < 0.0001).

The incubation time of MIRA, ranging from 2.5 to 20 min, was examined for detecting the *tlh*, *tdh*, and *trh* genes of *V. parahaemolyticus* (Figure 4A). MIRA for the *tlh* and *trh* genes began to show a positive band on the strip after an incubation time of 5 min, whereas MIRA for the *tdh* gene exhibited a positive band after 2.5 min. The band intensities of all genes increased in a time-dependent manner up to 20 min, with no significant differences observed between 15 and 20 min. Therefore, an incubation time of 15 min was selected for the MIRA step for all three genes. Previous studies using the RPA assay demonstrated that positive bands appeared at 8 min for *Salmonella* spp. and 20 min for *V. vulnificus* [32,35]. However, recent studies using the MIRA assay have reported that the positive test line could be observed after 5 min for *S. eriocheiris*, *A. baumannii*, and *Streptococcus agalactiae* [33,34,36]. Since the MIRA assay applies a different source of recombinase (*Streptomyces azure* recA, SC-recA) compared to the RPA assay that uses the RecA/Rad 51 ortholog of bacteriophage T4, T4 UvsX, this may result in different incubation times for amplification [31].

To determine the incubation time of the lateral-flow dipstick (LFD, Milenia Genline HybriDetect), the MIRA products of the *tlh*, *tdh*, and *trh* genes were inoculated onto the LFD with a time range from 1.5 to 10 min. As shown in Figure 4B, there were no significant differences in band intensities among the incubation times for all genes. Therefore, an incubation time of 1.5 min was selected to visualize the *tlh*, *tdh*, and *trh* genes with LFD strips. Based on the result of the incubation time for both the MIRA and LFD steps, the entire identification procedure to detect the *tlh*, *tdh*, and *trh* genes of *V. parahaemolyticus* could be accomplished within 20 min, including 3 min for the sample mixture procedure.

### 3.3. Evaluation of the Sensitivity and Specificity of the tlh, trh, and tdh Genes

**Figure 5:**
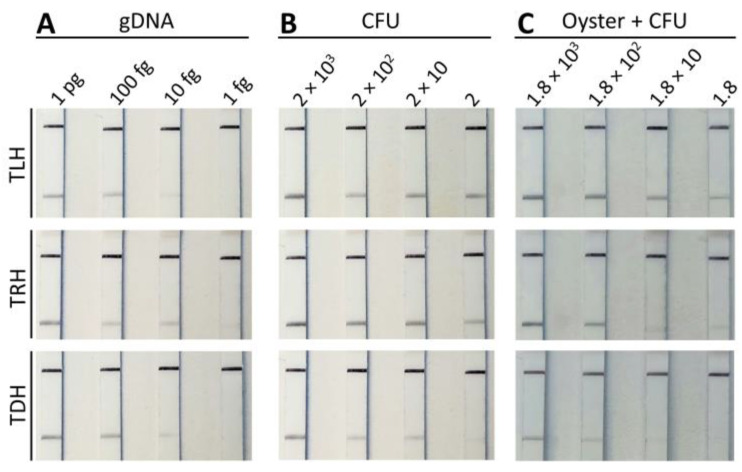
Evaluation of sensitivity of MIRA-LFD for the detection of *tlh*, *trh*, and *tdh* genes. The genomic DNA ((**A**) gDNA ranging from 1 pg to 1 fg), direct bacterial culture ((**B**) ranging from 2 × 10^3^ to 2 CFU), and seeded oyster ((**C**) gDNA ranging from 1 pg to 1 fg) were subjected to the MIRA-LFD assay. LFD strips exhibited an upper control line and a lower test line when the MIRA reaction was successfully accomplished.

The detection limits of the MIRA-LFD assay for the *tlh*, *tdh*, and *trh* genes were compared to those of PCR and qPCR assays. Figure 5 depicts four ten-fold serially diluted gDNA samples (ranging from 1 pg to 1 fg), bacterial cultures (ranging from 2 × 10^3^ CFU to 2 CFU), and seeded oysters (ranging from 1.8 × 10^3^ CFU to 1.8 CFU) using the three genes of *V. parahaemolyticus*. Positive test lines were observed with as little as 10 fg of gDNA, 2 CFU of bacterial culture, and 1.8 CFU of seeded oysters for the *tlh*, *tdh*, and *trh* genes. Previous studies on the detection of *V. parahaemolyticus* using RPA assays have reported detection limits ranging from 76 to 2 CFU of bacterial culture and 10 pg of genomic DNA [27,28,29,30]. More recently, MIRA assays for various pathogens have shown detection limits ranging from 760 to 6 CFU of bacterial culture and ranging from 97 pg to 64 fg of genomic DNA [31,33,34,36,37,38].

**Figure 6:**
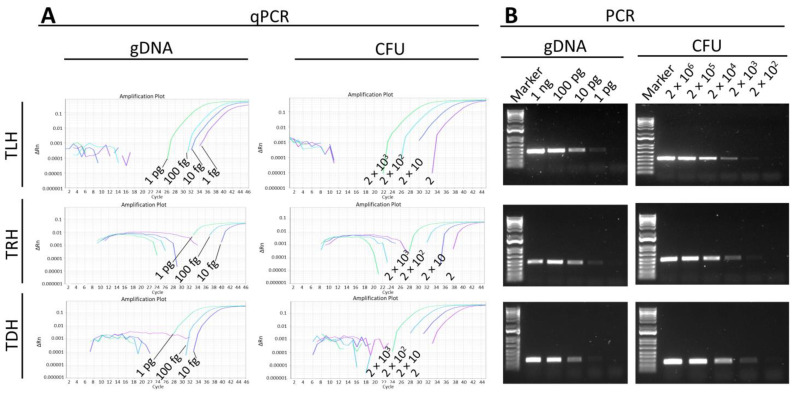
Evaluation of sensitivity of qPCR (**A**) and PCR (**B**) for the detection of *tlh*, *trh*, and *tdh* genes. Various concentrations of genomic DNA (gDNA) and direct bacterial culture (CFU) were subject to the detection of each gene using qPCR and PCR methods.

Similar detection limit results were obtained with the qPCR assay, which could detect as little as 10 fg of gDNA for the *tdh* and *trh* genes and 1 fg of the *tlh* gene (Figure 6A). The qPCR assay exhibited a detection limit of 2 CFU for all genes, which is consistent with a previous study that demonstrated that the qPCR assay could detect under 10 CFU [21]. However, the PCR assay was able to detect down to 1 pg of gDNA and 200 CFU of bacterial culture (Figure 6B). This result is in line with a previous study that reported a detection limit as low as 100 CFU [14]. The current MIRA-LFD assay for the *tlh*, *tdh*, and *trh* genes was as sensitive as qPCR and 100 and 200 times more sensitive than PCR in terms of gDNA and CFU, respectively.

**Figure 7:**
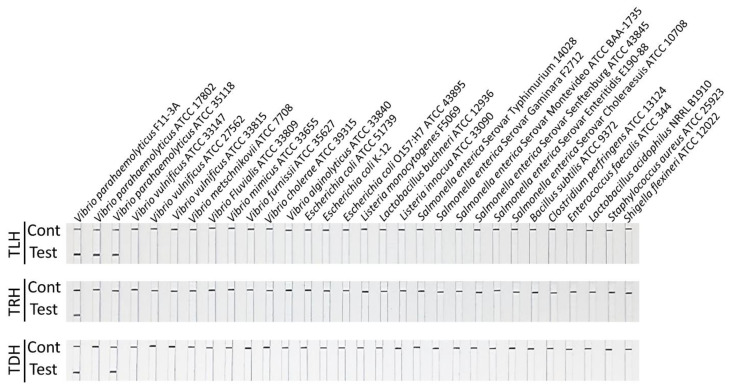
Evaluation of specificity of MIRA-LFD using genomic DNA from *Vibrio* and other foodborne pathogenic bacteria. The MIRA-LFD assay exhibited a positive test line for the positive control *V. parahaemolyticus* F11-3A for the *tlh*, *trh*, and *tdh* genes.

The specificity of MIRA-LFD for the *tlh*, *tdh*, and *trh* genes was determined using various bacteria listed in Table 1. As illustrated in Figure 7, the F11-3A strain exhibited positivity for all three genes. The 35118 strain showed positivity for the *tlh* and *trh* genes but tested negative for the *tdh* gene. In contrast, the 17802 strain exhibited positivity for the *tlh* gene but tested negative for the *tdh* and *trh* genes. All other *Vibrio* and foodborne pathogenic bacteria tested negative for all three genes. The results of the F11-3A, 35118, and 17802 strains regarding the *tlh*, *tdh*, and *trh* genes are consistent with previous studies [39,40]. Additionally, the PCR and qPCR assays exhibited concordant results for the three genes when compared with MIRA-LFD. Taken together with previous studies on MIRA-LFD, the current study indicates that MIRA-LFD is highly specific for target genes without cross-reactivity with other closely related bacteria [31,33,34].

### 3.4. Detection of tlh, trh, and tdh Genes from Oyster Samples

Fresh oysters were purchased from the local markets of four Northern Gulf coastal States in the U.S., and the oysters were enumerated to detect the *tlh*, *tdh*, and *trh* genes of *V. parahaemolyticus*. Each sample was prepared from ten blended oysters, and a total of 36 samples were analyzed to detect the three genes using the MIRA-LFD, PCR, and qPCR assays. As shown in Table 4, the species-specific *tlh* gene was detected using MIRA-LFD in 33.3%, 0%, 33.3%, and 16.6% of the oysters purchased in A, B, C, and D States, respectively. The same results were obtained using the qPCR assay. However, only 16.6% of samples from A State tested positive for the *tlh* gene using the PCR assay, and all samples from the other three States tested negative. Our results suggest that the MIRA-LFD assay is as sensitive as the qPCR assay, taken together with previous studies [31,38,41]. Interestingly, all oyster samples from all four States tested negative for the *tdh* and *trh* genes using the MIRA-LFD, PCR, and qPCR assays. This suggests that *V. parahaemolyticus* isolated from the environments may be less pathogenic compared to clinically isolated bacteria [10,11,12,13].

## 4. Conclusions

A rapid and simple MIRA-LFD assay has been developed to detect the species-specific *tlh* gene and the pathogenic-specific *tdh* and *trh* genes of *V. parahaemolyticus*. The careful selection of primers and probes enables the accurate amplification of the target regions of the genes by the MIRA-LFD assay, providing test results within 20 min. The assay for the three genes can detect as low as 10 pg of gDNA and 2 CFU of *V. parahaemolyticus*, demonstrating high specificity without the cross-detection of closely related bacteria. Further field tests indicated that MIRA-LFD has great potential for use in field conditions to detect the *tlh*, *tdh*, and *trh* genes of *V. parahaemolyticus* from oysters.

## Figures and Tables

**Table 1:** Bacteria used in this study and their amplification results for *tlh*, *trh*, and *tdh* genes using MIRA-LFD, PCR, and qPCR.

Bacteria	MIRA-LFD			PCR			qPCR		
	*tlh*	*trh*	*tdh*	*tlh*	*trh*	*tdh*	*tlh*	*trh*	*tdh*
*Vibrio parahaemolyticus* F11-3A	+	+	+	+	+	+	+	+	+
*Vibrio parahaemolyticus* ATCC 17802	+	−	−	+	−	−	+	−	−
*Vibrio parahaemolyticus* ATCC 35118	+	−	+	+	−	+	+	−	+
*Vibrio vulnificus* ATCC 33147	−	−	−	−	−	−	−	−	−
*Vibrio vulnificus* ATCC 27562	−	−	−	−	−	−	−	−	−
*Vibrio vulnificus* ATCC 33815	−	−	−	−	−	−	−	−	−
*Vibrio metschnikovii* ATCC 7708	−	−	−	−	−	−	−	−	−
*Vibrio fluvialis* ATCC 33809	−	−	−	−	−	−	−	−	−
*Vibrio mimicus* ATCC 33655	−	−	−	−	−	−	−	−	−
*Vibrio furnissii* ATCC 35627	−	−	−	−	−	−	−	−	−
*Vibrio cholerae* ATCC 39315	−	−	−	−	−	−	−	−	−
*Vibrio alginolyticus* ATCC 33840	−	−	−	−	−	−	−	−	−
*Escherichia coli* ATCC 51739	−	−	−	−	−	−	−	−	−
*Escherichia coli* K-12	−	−	−	−	−	−	−	−	−
*Escherichia coli* O157:H7 ATCC 43895	−	−	−	−	−	−	−	−	−
*Listeria monocytogenes* F5069	−	−	−	−	−	−	−	−	−
*Lactobacillus buchneri* ATCC 12936	−	−	−	−	−	−	−	−	−
*Listeria innocua* ATCC 33090	−	−	−	−	−	−	−	−	−
*Salmonella enterica* Serovar Typhimurium 14028	−	−	−	−	−	−	−	−	−
*Salmonella enterica* Serovar Gaminara F2712	−	−	−	−	−	−	−	−	−
*Salmonella enterica* Serovar Montevideo ATCC BAA-1735	−	−	−	−	−	−	−	−	−
*Salmonella enterica* Serovar Senftenburg ATCC 43845	−	−	−	−	−	−	−	−	−
*Salmonella enterica* Serovar Enteritidis E190-88	−	−	−	−	−	−	−	−	−
*Salmonella enterica* Serovar Choleraesuis ATCC 10708	−	−	−	−	−	−	−	−	−
*Bacillus subtilis* ATCC 9372	−	−	−	−	−	−	−	−	−
*Clostridium perfringens* ATCC 13124	−	−	−	−	−	−	−	−	−
*Enterococcus faecalis* ATCC 344	−	−	−	−	−	−	−	−	−
*Lactobacillus acidophilus* NRRL B1910	−	−	−	−	−	−	−	−	−
*Staphylococcus aureus* ATCC 25923	−	−	−	−	−	−	−	−	−
*Shigella flexineri* ATCC 12022	−	−	−	−	−	−	−	−	−

+: presence, −: absence.

**Table 2:** Primers and probes for the amplification of *tlh*, *trh*, and *tdh* genes using basic RPA and MIRA-LFD.

Assay	Names	Sequences (5′-3′)	Location	Amplicon Size (bp)
Basic RPA	VP_TLH_F1	AAAAACAATCACACTATTAACTGCATTACTCC		
	VP_TLH_R1	GTCAATGGTGAAGTAGCTACCATCTTCGTTTTT	6–231	226
	VP_TLH_R1-2	TTTAAATGAAACGGAGCTCCACCAGTAGCC	6–261	256
	VP_TLH_F2	CTCAGTTTAAGTACTCAACACAAGAAGAGAT		
	VP_TLH_R2	CTAAGTTGTTGCTACTTTCTAGCATTTTCT	869–1237	369
	VP_TLH_R2-2	TTGGATGCGTGACATCCCAGAACACAAACT	869–1180	312
	VP_TDH_F1	CTGTGAACATTAATGATAAAGACTATACAA		
	VP_TDH_R1	ATTACCAATATATTACCACTACCACTCTCATA	284–521	238
	VP_TRH_F1	ACTCTACTTTGCCTTCAGTTTGCTATTGGCTTC		
	VP_TRH-R1	GAAGTCGTGAAAATAGATTGACCGTGAACGCT	12–254	243
	VP_TRH-R1-2	AGGCGCTTAACCATTTTGAGCCTGAAGTCGTGA	12–277	266
	VP_TRH-F2	AGCGCCTATATGACGGTAAATATTAATGGAAAT		
	VP_TRH_R2	CATATGCCCATTTCCGCTCTCATATGCTTCGA	271–513	243
	VP_TRH-R2-2	TGACGAAATATTCTGGCGTTTCATCCAAATA	271–478	208
MIRA-LFD	VP_TLH_F1	AAAAACAATCACACTATTAACTGCATTACTCC		
	VP_PROBE	/56-FAM/TTCAGCGTCTGAAGTGATCAGCACGCAAGA/idSp/AACCAAACCTATACC/3SpC3/		
	VP_TLH_R1 _Biotin	Biotin-GTCAATGGTGAAGTAGCTACCATCTTCGTTTTT	6–231	
	VP_TDH_F1	CTGTGAACATTAATGATAAAGACTATACAA		
	VP_TDH_Probe	/56-FAM/AGCTTCAACATTCCTATGATTCTGTAGCTA/idSp/CTTTGTTGGTGAAGA/3SpC3/		
	VP_TDH_R1_Biotin	Biotin-ATTACCAATATATTACCACTACCACTCTCATA	284–521	
	VP_TRH_F1	ACTCTACTTTGCCTTCAGTTTGCTATTGGCTTC		
	VP_TRH_Probe	/56-FAM/TGAGCTACTATTTGTCGTTAGAAATACAAC/idSp/ATAAAAACTGAATCA/3SpC3/		
	VP_TRH_R1_Biotin	Biotin-GAAGTCGTGAAAATAGATTGACCGTGAACGCT	12–254	

**Table 3:** Primers and probes for the amplification of *tlh*, *trh*, and *tdh* genes using PCR and qPCR.

Assay	Names	Sequences (5′-3′)	Amplicon Size (bp)	Ref.
PCR	VPTLH_L	AAAGCGGATTATGCAGAAGCACTG		[14]
	VPTRH_R	GCTACTTTCTAGCATTTTCTCTGC	450	
	VPTRH-L	TTGGCTTCGATATTTTCAGTATCT		
	VPTRH-R	CATAACAAACATATGCCCATTTCCG	486	
	VPTDH-L	GTAAAGGTCTCTGACTTTTGGAC		
	VPTDH-R	TGGAATAGAACCTTCATCTTCACC	270	
qPCR	*tlh* 884 F	ACTCAACACAAGAAGAGATCGACCA		[21]
	*tlh* probe	/JOE/CGCTCGCGTTCACGAAACCGT/BHQ2		
	*tlh* 1091R	GATGAGCGGTTGATGTCCAA		
	*trh* 20F	TTGCTTTCAGTTTGCTATTGGCT		
	*trh* probe	/FAM/AGAAATACAACAATCAAAACTGA/MGBNFQ		
	*trh* 292R	TGTTTACCGTCATATAGGCGCTT		
	*tdh* 89F	TCCCTTTTCCTGCCCCC		
	*tdh* probe	/FAM/TGACATCCTACATGACTGTG/MGBNFQ		
	*tdh* 321R	CGCTGCCATTGTATAGTCTTTATC		
	IAC 46F	GACATCGATATGGGTGCCG		
	IAC Probe	/Cy5/TCTCATGCGTCTCCCTGGTGAATGTG/BHQ2		
	IAC 186R	CGAGACGATGCAGCCATTC		

**Table 4:** Comparison of amplification results for *tlh*, *trh*, and *tdh* genes among MIRA-LFD, PCR, and qPCR using oyster samples from four U.S. States.

States	*tlh*			*tdh*			*trh*		
	MIRA-LFD	PCR	qPCR	MIRA-LFD	PCR	qPCR	MIRA-LFD	PCR	qPCR
A	2/6 (33.3% *)	1/6 (16.6%)	2/6 (33.3%)	0/6 (0%)	0/6 (0%)	0/6 (0%)	0/6 (0%)	0/6 (0%)	0/6 (0%)
B	0/6 (0%)	0/6 (0%)	0/6 (0%)	0/6 (0%)	0/6 (0%)	0/6 (0%)	0/6 (0%)	0/6 (0%)	0/6 (0%)
C	2/6 (33.3%)	0/6 (0%)	2/6 (33.3%)	0/6 (0%)	0/6 (0%)	0/6 (0%)	0/6 (0%)	0/6 (0%)	0/6 (0%)
D	3/18 (16.6%)	0/18 (0%)	3/18 (16.6%)	0/18 (0%)	0/18 (0%)	0/18 (0%)	0/18 (0%)	0/18 (0%)	0/18 (0%)

* The percentage indicates positive results from the oyster samples examined.

## Data Availability

Data are contained within the article.
